# Tissue-derived small extracellular vesicles in cancer diagnosis and prognosis: current insights and future directions

**DOI:** 10.1186/s12964-026-02894-0

**Published:** 2026-04-29

**Authors:** Benjie Li, Qi Wang, Baokun Fan, Bairen Pang, Yong Li, Junhui Jiang

**Affiliations:** 1 Health Science Center, Ningbo University, Ningbo, Zhejiang 315211 China; 2 Translational Research Laboratory of Urology, The First Affiliated Hospital of Ningbo University, Ningbo, Zhejiang 315010 China; 3 Ningbo Clinical Research Center of Urology Diseases, The First Affiliated Hospital of Ningbo University, Ningbo, Zhejiang 315010 China; 4 Zhejiang Engineering Research Center of Innovative Technologies and Diagnostic and Therapeutic Equipment for Urinary System Diseases, Ningbo, Zhejiang 315010 China; 5 St George and Sutherland Clinical Campuses, School of Clinical Medicine, UNSW Sydney, Kensington, NSW 2052 Australia; 6 Cancer Care Centre, St George Hospital, Kogarah, NSW 2217 Australia; 7 Department of Urology, Ningbo First Hospital, The First Affiliated Hospital of Ningbo University, Ningbo, Zhejiang 315600 China

**Keywords:** Tissue-derived small extracellular vesicles, Tumour microenvironment, Cancer diagnosis, Cancer prognosis, Extracellular vesicles

## Abstract

**Background:**

Cancer remains one of the leading causes of human death worldwide. Small extracellular vesicles (sEVs) are nanoscale particles that play important roles in intercellular communication in cancer and represent a valuable source for cancer biomarker discovery. Tissue-derived small extracellular vesicles (Ti-sEVs) are a specific subtype of sEVs and have attracted increasing attention as an emerging research area in cancer diagnostics and therapeutics. Recent progress has highlighted the distinct advantages of Ti-sEVs, including greater cancer specificity, minimal exogenous contamination, enhanced potential for cancer biomarker discovery and understanding tumour microenvironment (TME) dynamics.

**Methods:**

This review summarises recent advancements in the preparation, isolation, and characterization of Ti-sEVs. It further discusses current knowledge of their biological functions within the TME and critically evaluates their emerging application in cancer diagnosis and prognosis.

**Conclusion:**

Ti-sEVs offer unique opportunities for cancer biomarker discovery and translational research. However, key challenges remain, including tissue processing variability, methodological standardization, and technical barriers to clinical implementation. Addressing these issues will be essential to realize the full potential of Ti-sEV-based diagnostics and therapeutics and to guide future research in this rapidly evolving field.

## Introduction

Cancer remains a significant global health challenge. In 2019, the World Health Organization (WHO) reported cancer as the primary or secondary leading cause of death in 112 out of 183 countries, with a particularly high impact on individuals under the age of 70. By 2025, an estimated 2.04 million new cancer cases and 618,000 cancer-related deaths are projected to occur in the United States alone [1]. This figure is projected to rise to 28.4 million new cases annually by 2040, driven by aging populations, lifestyle changes, and environmental factors [1, 2]. In China, cancer incidence and mortality rates are increasing rapidly, contributing significantly to the global cancer burden [3, 4]. These alarming trends underscore the urgent need for advancements in cancer screening, new early detection, and diagnostic strategies to mitigate the growing impact of this disease on global health.

Despite technological advancements, early and accurate cancer diagnosis remains challenging due to tumour heterogeneity, sampling bias, and limitations of existing diagnostic tools [5]. Traditional tissue biopsy, regarded as the gold standard for cancer diagnosis, is invasive and carries the risk of sampling errors [5, 6]. Furthermore, tumour heterogeneity can lead to incomplete diagnostic insights. Consequently, research into alternative diagnostic methods is essential to address these gaps and improve early detection rates [7].

Extracellular vesicles (EVs) have emerged as promising biomarkers for cancer diagnosis. These lipid bilayer–delimited particles are secreted by nearly all cell types and carry molecular cargo, including DNA, RNA, and proteins that reflect the state of their cells of origin. Evidence has shown that EVs mediate intercellular communication and mirror physiological and pathological states. In line with MISEV2023, EV subtypes are preferably described using operational terms: “sEVs” and “lEVs” denote EV preparations enriched predominantly in vesicles ≤ 200 nm and > 200 nm, respectively, while acknowledging that these size-based designations depend on the characterization method used and that overlap between EV populations may occur. Among these EV subtypes, sEVs have attracted particular interest in cancer biomarker research [8–12]. Tissue-derived sEVs (Ti-sEVs), obtained from tumor tissues via invasive procedures such as surgical excision or biopsy, offer high-quality molecular information directly representative of the tumor microenvironment (TME) [13, 14].

Although the direct acquisition of Ti-sEVs require invasive procedures, these vesicles can subsequently enter bodily fluids (e.g., blood and urine) and circulate systemically, thereby offering a bridge between tissue-based analysis and liquid biopsy. Ti-sEVs released into the TME are transported via bodily fluids, carrying tumor-associated biomolecules that enhance the molecular specificity of liquid biopsies [13–16]. Studying Ti-sEVs not only elucidates the biogenesis and functional properties of circulating sEVs but also improves the diagnostic accuracy and clinical utility of liquid biopsy platforms.

Liquid biopsy, including sEVs, has gained prominence as a non-invasive diagnostic modality that detects tumour-derived components in biofluids, such as plasma, urine, and saliva. It enables applications ranging from early screening and monitoring of therapeutic response to the assessment of tumour heterogeneity and recurrence [17–22]. In liquid biopsy, body fluid-derived sEVs (BF-sEVs) are widely studied as tumor biomarkers. However, their clinical translation is hampered by heterogeneity, low abundance, and co-isolation of non-tumour EVs [13, 14, 23–25].

Ti-sEVs represent a valuable reference material that can mitigate these limitations. As tumour-specific vesicles directly isolated from the TME, Ti-sEVs exhibit reduced contamination from non-malignant sources and offer higher tumour specificity than BF-sEVs. Their molecular profiles can inform the selection of tumour-specific markers for liquid biopsy assays, thereby improving detection sensitivity and specificity [13–16]. Consequently, Ti-sEVs are poised to play an essential role in refining liquid biopsy-based cancer diagnostics and personalised medicine.

The molecular composition of Ti-sEVs provides a precise snapshot of the TME, including genetic alterations, signalling pathways, and immune modulation mechanisms, with minimal contribution from non-tumour sources [16, 26]. This high level of tumor specificity positions Ti-sEVs as invaluable tools for early cancer detection, monitoring tumour progression, and the development of targeted therapies [16, 26, 27]. As research advances, Ti-sEVs are increasingly recognized for their potential to identify tumour-specific biomarkers, enabling the development of tailored therapeutic strategies. These vesicles hold significant potential for enhancing cancer diagnosis and prognosis, addressing the challenges posed by tumour heterogeneity, and offering molecular insights to guide clinical decision-making.

This review provides a comprehensive overview of current methodologies for the isolation and characterization of Ti-sEVs, with an emphasis on their unique potential in cancer diagnosis and prognosis. Additionally, the limitations and challenges associated with Ti-sEVs are also discussed, along with their prospective applications in improving early cancer detection, monitoring disease progression, and advancing personalized cancer treatment.

## Isolation techniques for Ti-sEVs

The isolation of high-purity sEVs is crucial for ensuring accurate research and enabling their practical applications. Biological samples such as human tissues, body fluids, and cell culture media are typically rich in free proteins, cellular debris, and non-vesicular particles [8, 28], posing significant challenges to achieving sEV purification. These contaminants can compromise downstream analyses, obscure the detection of sEV-specific markers, and impede a thorough understanding of sEVs' biological functions. Addressing these challenges is essential for advancing sEV research and realise their potential in diagnostics and therapeutics. Notably, the isolation of Ti-sEVs remains an understudied area, necessitating further research to fully leverage their unique insights into the TME remodeling, angiogenesis, tumor invasion and metastasis, and immune evasion. Different sEVs isolation methods with advantages and limitations are summarized in Table 1.

**Table 1:** Different isolation methods for different sources derived sEVs

Method	Mechanisms	Advantages	Limitations	EVs Type	References
Ultracentrifugation (UC)	Differential or density gradient centrifugation based on size and density of EVs	Gold standard, widely used, no reagents required	Time-consuming, requires expensive equipment, risk of co-isolation of contaminants	CCM-sEVs; BF-sEVs; Ti-sEVs	[29–31]
Size-Exclusion Chromatography (SEC)	Separation based on size differences in a porous matrix	Gentle on EVs, retains bioactivity, high purity	Limited scalability, requires pre-concentration of samples	CCM-sEVs; BF-sEVs;Ti-sEVs	[29–33]
Immunomagnetic Beads	Antibody-coated magnetic beads specifically bind EV surface markers	High specificity, fast, easy to automate	Requires specific antibodies, low yield, risk of antibody detachment	CCM-sEVs; BF-sEVs;	[34–36]
Microfluidic Chip Technology	EV isolation using size, charge, or affinity-based separation within a microfluidic device	Minimal sample requirement, fast, highly customizable	Expensive, complex setup, may require specialized training or equipment	CCM-sEVs; BF-sEVs;	[35, 37, 38]
Asymmetric-Flow Field-Flow Fractionation (AF4)	Separation based on hydrodynamic forces in a flow field	High-resolution separation, gentle on EVs	Expensive equipment, requires expertise, limited accessibility	CCM-sEVs; BF-sEVs;	[37, 39, 40]
EXODUS	Detection and quantification of EVs as they pass through a nanopore	High yield, purity, and scalability	Novel method, limited commercial availability, may require specialized equipment	CCM-sEVs; BF-sEVs;	[41]

*Abbreviations: AF4* asymmetric flow field-flow fractionation, *BF-sEVs* body fluid-derived sEVs, *CCM-sEVs* cell culture medium-derived sEVs, *SEC* size exclusion chromatography, *Ti-sEVs* tissue-derived sEVs, *UC* ultracentrifugation

### Current Ti-sEVs pretreatment process

**Fig. 1:**
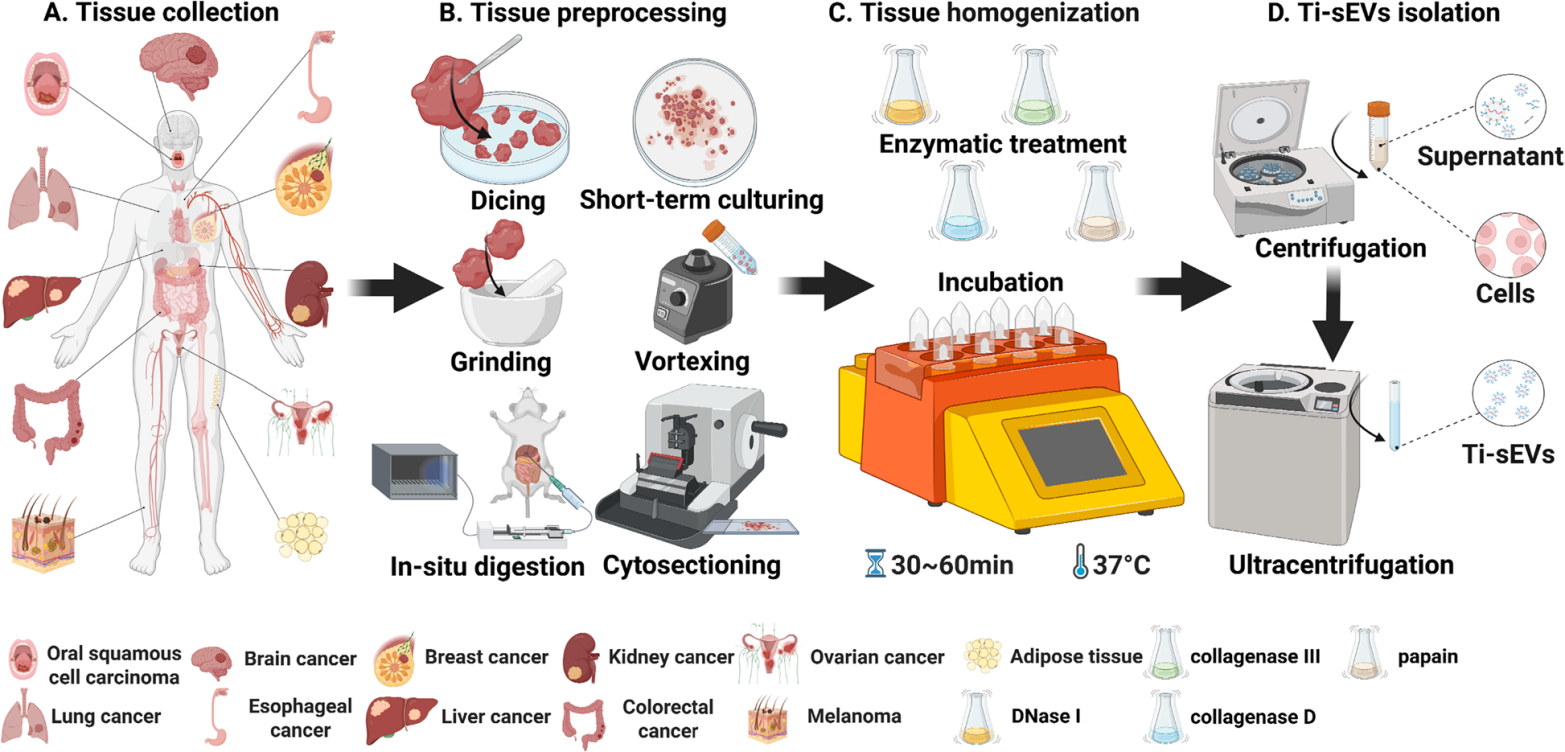
Comprehensive summary of Ti-sEV isolation workflow from various tissue types. **A** Ti-sEVs can be isolated from a wide range of tissues, including brain, lung, esophageal, liver, breast, kidney, colorectal, ovarian, oral squamous cell carcinoma, adipose tissue, and melanoma tissues. **B** Tissue handling involves techniques such as dicing, vortexing, grinding, and manual mincing, with options for short-term culture or in-situ digestion using animal models. **C** Enzymatic treatment employs enzymes such as DNase I, collagenase (types III and D), and papain. Tissue samples are incubated at 37 °C for 30–60 min to create a single-cell suspension or prepare the tissue supernatant. **D** Pre-treated tissue samples are centrifuged to separate supernatant and single cells. Ti-EVs are isolated using ultracentrifugation from the processed supernatant

sEV isolation from tissue specimens presents distinctive challenges compared with biofluids, primarily because the complex extracellular matrix (ECM) both restricts vesicle release and complicates their subsequent recovery. The ECM not only forms a highly cross-linked structural scaffold but also actively sequesters sEVs through specific interactions between EV surface molecules including integrins, fibronectin, CD44 and matrix components such as collagen, fibronectin and laminin, thereby markedly limiting their effective release and interstitial mobility [42, 43]. To overcome this barrier, tissue-derived sEV protocols commonly incorporate collagenase-based enzymatic digestion. However, studies in brain tissue have shown that even mild collagenase treatment can induce artificial proteolytic cleavage of key EV-associated membrane proteins such as Alix, CD9, CD81, GM130, thereby distorting canonical marker profiles and potentially compromising downstream quantitative and functional analyses of tissue-derived sEVs [44]. Consequently, recent guidelines, such as MISEV2023, strongly emphasize the careful optimization and transparent reporting of tissue dissociation protocols in order to balance sEV yield with the preservation of vesicle integrity [8]. Recent advancements have focused on developing specialized protocols to overcome these obstacles, incorporating optimized tissue processing techniques, enzymatic dissociation strategies, and tailored purification methods to enhance sEV yield and purity A key challenge in isolating Ti-sEVs lies in tissue homogenization process, which must achieve high vesicle yield while preserving tissue cell integrity to minimize the release of intracellular vesicles and other non-sEV components that could confound downstream analyses. A comprehensive overview of the Ti-EV isolation workflow from various tissue types is illustrated in Fig. 1. Consequently, appropriate pretreatment of tissues prior to Ti-sEV isolation is essential to address these challenges and ensure the recovery of highly pure and biologically representative vesicles.

To address these challenges, particular attention must be given to the initial steps of tissue processing, especially the homogenisation and enzymatic dissociation procedures. The tissue homogenization pretreatment process typically involves two key steps: first, increasing the tissue surface area; and second, dissociating the tissue using suitable enzymatic treatments [13, 14]. The freezing–thawing process can induce cellular stress and membrane compromise, leading to the release of intracellular vesicles that contribute to the increased particle yield observed in frozen samples. To address this, the study by Shen et al. [45] provides crucial insights. They systematically compared two − 80 °C preservation strategies: tissue frozen and lysate frozen, against the fresh-tissue method. While both frozen approaches yielded higher particle counts, which may partially reflect stress-induced release, the critical finding lies in the downstream characterization of the isolated Ti-sEVs. Despite the potential contribution of freezing-associated vesicles, the Ti-sEVs obtained from both frozen protocols were demonstrated to be functionally and molecularly intact. Specifically, they were virtually indistinguishable from fresh-tissue-derived sEVs in terms of size distribution, morphology, proteomic profile, miRNA repertoire, and functional activity [45]. Therefore, while the initial yield increase likely includes a component of stress-related particles, the rigorous isolation and characterization protocols effectively enrich for a population of bona fide sEVs whose native properties are preserved. This supports the conclusion that − 80 °C cryopreservation introduces minimal perturbation to the functionally relevant Ti-sEVs, validating it as a practical strategy for biobanking [45]. Nonetheless, to mitigate the variable impact of freeze–thaw stress, the use of fresh tissues remains the preferred option when logistically feasible to ensure maximum experimental consistency [14].

Current studies have shown that a wide range of tissues, including brain, liver, ovarian, kidney, colorectal, lung, esophageal, adipose tissue, oral tissues and melanoma tissues can be used to isolated Ti-sEVs [14, 29, 46–52] (Fig. 1A). Various methods have been explored for tissue handling and Ti-sEVs isolation, including tissue dicing [13, 14, 53, 54], short-term culture [47], grinding [55, 56], vortexing [57], in-situ digestion in animal models [30, 58] and cytosectioning [59] as shown in Fig. 1B. In practice, tissue dicing involves gently slicing specimens on ice into small, uniform fragments (e.g., 2 × 2 × 2 mm cubes) to facilitate enzyme penetration while preserving ECM integrity and cell–cell junctions as much as possible. This approach offers the advantage of yielding a higher amount of sEVs with reduced contamination from intracellular vesicles. However, even with this gentler approach, a certain degree of mechanical damage to cells remains unavoidable [13, 14, 53, 54]. On the other hand, mincing typically involves more aggressive and disruptive mechanical dissociation, using repeated scalpel cutting or mechanical choppers to break the tissue down into a pulp- or paste-like consistency, which can severely damage the ECM and cell–cell junctions and may compromise overall tissue integrity. Nevertheless, when mincing is followed by short-term culture to isolate Ti-sEVs from the supernatant, it offers the advantage of facilitating direct sEV release into the culture medium during cultivation. This approach simplifies the collection process by generating a more concentrated sEV sample and bypassing the need to extract sEVs directly from complex tissue matrices, thereby streamlining subsequent separation and analysis steps [47]. However, a key limitation is that sEVs obtained through culture may not fully represent the native in vivo vesicle population, potentially altering functional interpretations [13]. Using a mechanical grinding method to homogenize tissue offers the advantage of yielding a sufficient quantity of sEVs [55, 56, 60]. However, this approach carries the drawback of an increased risk of cell rupture, which may lead to contamination with intracellular vesicles, potentially compromising the purity of the isolated sEVs [13]. Vortexing tissues offers several advantages for EV isolation, including its simplicity, ease of operation, and increased EV yield. However, a notable drawback is that vortexing can cause cell damage and apoptosis, leading to an increase in cellular debris and potentially compromising sEV purity [57]. In situ digestion of tissues using animal models offers the advantage of flushing out blood through in situ perfusion, which helps prevent cell rupture and the formation of coagulation products. However, this method still necessitates subsequent tissue mincing, and the limited availability of relevant literature makes the procedure complex and challenging to standardize [30, 58]. Cryosectioning offers several advantages over traditional surgical slicing for isolating EVs from tissues, particularly by improving sEV yield and experimental consistency. By cutting tissues into thinner sections at low temperatures, cryosectioning facilitates more efficient enzymatic digestion, minimizing tissue and sEV loss associated with manual cutting [59]. Additionally, the standardized slicing process reduces human error, enhancing the reproducibility and consistency of experimental results. In addition, low-temperature treatment also helps preserve cellular integrity, limiting the release of apoptotic bodies and intracellular contents that could interfere with sEV analysis [59]. This preservation is crucial, as tissue microenvironments and cellular activity degrade rapidly post-dissection, impacting sEV purity and quality. However, cryosectioning is not without its drawbacks. The process can still cause cellular damage, leading to contamination from leaked intracellular contents. This contamination may overestimate sEV yield, obscure biomarkers, and compromise the sensitivity of downstream analyses such as proteomics and functional assays. Despite these challenges, cryosectioning remains a valuable technique for improving the efficiency and reliability of sEV extraction from tissues, offering a balance between yield, purity, and consistency. Consequently, tissue slicing remains widely used due to its ability to maintain ECM integrity and cell–cell junctions, thereby supporting the isolation of structurally intact sEVs. In practice, fresh tumour tissue is gently cut on ice with a scalpel into approximately 2 × 2 × 2 mm fragments in culture medium (about 1 ml medium per 0.2 g tissue), which are then transferred at roughly 0.2 g tissue in 2 ml RPMI-1640 per well for subsequent enzymatic digestion and EV release. [13, 14, 53, 54]. Emerging techniques such as cryosectioning present promising alternatives, though conclusive comparative evidence is still needed to establish optimal methodologies.

Following tissue handling, enzymatic digestion is performed to disrupt the ECM and facilitate sEV release (Fig. 1C). It is crucial to optimize factors such as buffer composition, pH, and osmotic pressure to maintain cellular stability and prevent degradation. For example, after tissue dicing, the tissue pieces can be incubated in an RPMI-1640–based digestion buffer containing collagenase D (2 mg/mL) and DNase I (40 U/mL) at 37 °C for 30 min to gently release tissue-derived EVs from the matrix while preserving vesicle integrity [13]. Specific enzymes are required to target ECM components and intercellular junctions. For instance, in mouse cochlear tissue, researchers evaluated various enzymes, including collagenase D with DNase I, collagenase III, and papain. The study identified 3 U of collagenase D and 40 U of DNase I as the most effective combination for EV isolation [61]. Collagenase D is widely used for tissue digestion, collagenase D cleaves peptide bonds in collagen, facilitating ECM breakdown and the release of cells and EVs into suspension [31, 32, 61]. DNase I is an ideal for tissue digestion and single-cell preparation, DNase I degrades free DNA released from dead cells, reducing extracellular DNA-induced aggregation without triggering apoptosis [61]. This enzymatic combination not only improves sEV yield but also ensures the purity of isolated vesicles, providing a reliable approach for sEV isolation from complex tissues.

In another study, using melanoma tissue as a model, the processing started with storing the tissue in phosphate-buffered saline on ice to maintain its structural and functional integrity. The tissue was then carefully diced into uniform 2 × 2 × 2 mm pieces using a scalpel to ensure consistent enzymatic digestion. Subsequently, the tissue fragments were incubated at 37 °C for 30 min in a cell culture medium containing collagenase D and DNase I, promoting effective enzymatic breakdown. After digestion, the mixture is passed through a 70 μm pore-size filter to remove larger tissue fragments and impurities. The filtered solution undergoes sequential centrifugation steps and ultracentrifugation steps to separates tissue derived single-cells or Ti-sEVs as shown in the Fig. 1D. Digested and filtered samples were initially centrifuged at 300 g force for 10 min to separate cells and large debris, followed by centrifugation at 2,000 g force for 20 min to clear residual tissue debris. The resulting supernatant was then processed further to isolate EVs subtypes, including large EVs (lEV; typically > 200 nm) and sEVs [29]. This protocol facilitates efficient EV extraction and purification while minimizing contamination from cellular components.

This optimized pretreatment process ensures efficient isolation of tissue-derived sEVs while minimizing contamination with intracellular vesicles. The choice of enzymes, combined with careful tissue handling, is key to achieving high-quality EV preparations for downstream applications. A comprehensive summary of representative tissue preprocessing approaches, enzymatic digestion conditions, isolation workflows, and key methodological considerations across different tissue types is provided in Table 2.

**Table 2:** Comparison of tissue preprocessing and isolation strategies for Ti-sEVs

Tissue/Cancer type	Preprocessing method	Enzymatic digestion	Ti-sEV isolation method	Key considerations	References
Metastatic melanoma tissue	Tumour tissue was carefully dissected to increase exposed surface area and incubated in culture medium for 30 min	DNase I + collagenase D	70 µm filtration → 300 × g, 10 min → 2,000 × g, 20 min → 16,500 × g, 20 min or 118,000 × g, 2.5 h	Tissue dicing: This approach preserves tissue architecture better than aggressive homogenisation, but cutting-associated mechanical injury may still induce release of intracellular vesicles. Enzymatic artefacts and recovery efficiency are highly dependent on tissue type, ECM composition, and digestion conditions, leading to a recurrent yield–purity trade-off	[14]
Cochlear tissue (neonatal mouse)	Cochleae were dissected and cut into ~ 2 mm pieces prior to dissociation	Collagenase D + DNase I/collagenase III/papain; optimised: 3 U collagenase D + 40 U DNase I	300 × g, 10 min → 2,000 × g, 20 min → 0.22 µm filtration → 16,500 × g, 20 min → UC (110,000 × g, 2 h) or SDGU (180,000 × g, 3 h → 110,000 × g, 2 h) or SEC (fractions F1–F6; 100 kDa MWCO concentration)		[61]
Clear cell renal cell carcinoma (ccRCC) tissue	Primary kidney tissue was dissociated and clarified before downstream purification	Type 3 collagenase	300 × g, 5 min → 2,000 × g, 20 min → 10,000 × g, 30 min → 100,000 × g, 90 min → sucrose gradient (180,000 × g, 180 min) → 100,000 × g, 90 min wash		[62]
Bladder cancer tissue	Approximately 1 g of tissue was cut into 2–3 mm pieces and washed with PBS	Collagenase D (2 mg/mL) + DNase I (40 U/mL), 37 °C, 30 min	1,000 × g, 10 min → 10,000 × g, 20 min → 0.45 µm PVDF filtration → 10 kDa MWCO concentration to 0.5 mL → qEV original 70 nm SEC → pooled fractions 7–10 → 10 kDa MWCO concentration to 100 µL		[63]
OSCC tissue	Tissue samples were gently minced into < 1 mm fragments using fine scissors	Collagenase IV (1 mg/mL) + DNase I (0.1 mg/mL), 37 °C, 500 rpm, 60 min	70 µm filtration → 300 × g, 10 min → 2,000 × g, 20 min → 10,000 × g, 45 min → 120,000 × g, 70 min		[64]
Epithelial ovarian cancer tissue	Tissue was thawed on ice, washed, trimmed, and mechanically fragmented to < 2 mm	Type I collagenase (1 mg/mL) + DNase I (0.2 mg/mL), 37 °C, 30 min	300 × g, 10 min × 2 → 70 µm filtration → 2,000 × g, 15 min → 10,000 × g, 30 min → 100,000 × g, 90 min → SEC (fractions 3–7) → 100,000 × g, 90 min		[65]
Brain tissue	Frozen tissue was weighed and briefly sliced on dry ice before digestion	Collagenase III (75 U/mL), 37 °C; 20 min (human) or 15 min (macaque/mouse)	300 × g, 10 min → 2,000 × g, 15 min → 0.22 µm filtration → 10,000 × g, 30 min → SDGU or SEC + UC/UF		[44]
Ovarian clear cell carcinoma tissue	Freshly resected tissue was incubated in serum-free medium for 3 h before vesicle recovery	NR	0.22 µm filtration → 2,000 × g, 30 min → 16,000 × g, 30 min → 100,000 × g, 70 min	Short-term culture: This strategy minimises direct mechanical disruption, but ex vivo incubation may shift the native in vivo sEV profile. Vesicle yield and cargo composition are strongly influenced by culture duration and medium conditions	[66]
Colorectal cancer tissue	Tissues were washed, cut, and mechanically homogenised in cold PBS	NR	4,000 × g, 30 min → 12,000 × g, 30 min → 110,000 × g, 2 h → 30% sucrose cushion → 110,000 × g, 2 h → PBS wash → 110,000 × g, 2 h	Grinding: Mechanical homogenisation may enhance particle release from dense tissues, but it also markedly increases the risk of cell rupture, intracellular vesicle release, and contamination, thereby favouring yield over specificity	[67]
Breast cancer tissue	Tissue specimens were cut into 300 µm sections using a freezing microtome	Papain Dissociation System, 37 °C, 10–15 min	70 µm filtration → 300 × g, 10 min → 2,000 × g, 10 min → 10,000 × g, 20 min → 0.22 µm filtration → 150,000 × g, 2 h → Exosupur columns → UF	Cytosectioning: Uniform sectioning improves reproducibility and enzymatic accessibility, but the workflow requires specialised equipment and strict low-temperature control. Subsequent enzyme-assisted release may still introduce tissue-dependent variability	[68]
Myometrial tissue	A small frozen tissue slice (~ 200 mg) was prepared on dry ice	Human tumour dissociation enzyme mix (enzyme H + R + A), 37 °C, 10–15 min	70 µm filtration × 2 → 300 × g, 10 min → 2,000 × g, 10 min → 10,000 × g, 20 min → 0.22 µm filtration → 150,000 × g, 2 h → Exosupur columns → 100 kDa UF		[53]
Heart, kidney and stomach tissues	Tissues were snap-frozen and cut into 100–300 µm sections in a cryostat	Tissue dissociation reagents, 37 °C, 10–30 min	70 µm filtration → 300 × g, 10 min → 3,000 × g, 10 min → 10,000 × g, 10 min → 150,000 × g, 2 h → 0.22 µm filtration → SEC → UF		[59]
Brain tissue	Frozen grey matter (~ 600 mg) was placed in cold PBS, gently teased, and vortexed	NR	300 × g, 10 min → 1,200 × g, 10 min × 2 → 0.2 µm filtration → 10,000 × g, 30 min × 2 → 22,000 × g, 22 h	Vortexing: Although operationally simple, vortexing may generate pronounced mechanical artefacts, including cellular disruption, debris release, and intracellular vesicle contamination, thereby limiting specificity for bona fide sEVs	[57]
Liver tissue (mouse)	Portal vein cannulation and two-step liver perfusion were performed in situ	Collagenase IV, optimised at 1–2 mg/mL for 7–8 min	50 × g, 10 min → 300 × g, 10 min → 2,000 × g, 20 min → 10,000 × g, 70 min → 100,000 × g, 70 min → PBS wash → 100,000 × g, 70 min	In situ digestion (animal models): Perfusion-based preprocessing can reduce blood-derived interference and improve enzyme delivery, but the workflow is technically demanding, model-dependent, and highly sensitive to digestion and perfusion parameters	[58]
Heart tissue (animal model)	Cardiac tissue was collected after ice-cold PBS perfusion; apex was minced for 30 s at 4 °C in 0.9% NaCl (100 µL/100 mg tissue)	NR	400 × g, 15 min × 2 → 20,500 × g, 45 min → precipitation buffer overnight at 4 °C → 10,000 × g, 1 h → 100,000 × g, 90 min		[30]

*Abbreviations: ccRCC* clear cell renal cell carcinoma, *ECM* extracellular matrix, *MWCO* molecular weight cut-off, *NR* not reported, *OSCC* oral squamous cell carcinoma, *PVDF* polyvinylidene fluoride, *SDGU* sucrose density gradient ultracentrifugation, *SEC* size-exclusion chromatography, *sEVs* small extracellular vesicles, *UC* ultracentrifugation, *UF* ultrafiltration

### Current Ti-sEVs isolation methods

The success of Ti-sEV isolation depends heavily on the effectiveness of both the pretreatment process and the subsequent isolation techniques. MISEV2023 recommends that, for complex sources such as solid tissues, at least two successive low-speed centrifugation and/or filtration steps should be performed after tissue digestion or homogenisation to remove cells and large debris, followed by sequential combinations of complementary methods (e.g., differential or density-gradient ultracentrifugation with SEC or AF4) to maximise the specificity and purity of EV preparations while maintaining acceptable recovery [8].

Ultracentrifugation (UC) is a widely employed method for isolating Ti-sEVs, with specific protocols adapted for different tissue types, such as myometrial and cochlear tissues. In the case of myometrial tissue from pregnant women [53], the process begins by centrifuging the supernatant obtained after pretreatment at 10,000 g force for 20 min at 4 °C to precipitate larger particles. The pellet is then filtered through a 0.22 micron filter to reduce cellular debris. The supernatant is subsequently subjected to ultracentrifugation at 150,000 g force for 2 h at 4 °C to collect the sEVs pellet. For cochlear tissue [61], the supernatant is initially centrifuged at 16,500 g force for 20 min at 4 °C to remove larger vesicles, with the pellet being discarded. The remaining supernatant is further processed through ultracentrifugation at 110,000 g force for 2 h at 4 °C to precipitate Ti-sEVs. After centrifugation, the Ti-sEVs pellet is resuspended in 150 μL of DPBS and stored at −80 °C for subsequent analyses. These protocols highlight the versatility and efficiency of ultracentrifugation in isolating sEVs from various tissue types, ensuring high specificity and purity in the final preparations.

Size exclusion chromatography (SEC) is another widely employed method for isolating Ti-sEVs. This technique separates vesicles based on their hydrodynamic diameter as they pass through a porous stationary phase [69]. Following tissue pretreatment, the processed sample is applied to an SEC column. For example, using a qEVoriginal-35 nm SEC column (Izon Science), the column is first equilibrated with 20 mL of DPBS to condition the matrix. The sample, concentrated to 500 μL, is then carefully loaded onto the column. Once the sample has completely entered the column and the liquid flow stops, additional DPBS is added to facilitate separation. During fraction collection, the initial 3 mL of eluate is discarded to remove non-vesicular components. Subsequently, first three or six fractions are collected with 500 μL in each fractions. These fractions are then concentrated to approximately 150 μL using a 100 kDa molecular weight cut-off filter. This SEC method is particularly effective at preserving the structure and integrity of Ti-sEVs throughout the isolation process, making it a reliable technique for sEV preparation [33, 61].

Density gradient centrifugation is a widely employed technique for isolating sEVs, often utilizing the sucrose density gradient method. The process begins with preparing sucrose solutions at concentrations of 2.5 M, 1.3 M, and 0.6 M, which are carefully layered into an ultracentrifuge tube in decreasing density order to form a sucrose density gradient cushion. After tissue digestion, the supernatant obtained from centrifugation at 16,500 × g is gently added to the sucrose cushion, and the total volume is adjusted to 7.5 mL with DPBS. The sample is centrifuged at 180,000 g force for 3 h at 4 °C to separate components based on their densities. After centrifugation, the top supernatant is removed, and different fractions are collected and then diluted with DPBS to centrifuging at 110,000 g force for 70 min at 4 °C to precipitate Ti-sEVs. Rossella et al. proposed an alternative approach that combines differential centrifugation with density gradient centrifugation, replacing the traditional sucrose gradient with iodixanol. This method successfully isolated six distinct Ti-sEVs subpopulations from tissues, enabling size-based separation and demonstrating wide applicability across various tissue types [14].

Additionally, studies have compared traditional separation methods, including density gradient centrifugation combined with UC, SEC combined with UC, and SEC combined with ultrafiltration (UF), assessing their impact on the purity and yield of Ti-sEVs using sEV miRNA sequencing. Among these approaches, SEC combined with UC was identified as the most effective, offering superior yield and purity. Consequently, it is recommended as the preferred technique for the isolation of Ti-sEV isolation [13, 14].

Ti-sEVs have been successfully isolated from a range of human and animal tissues, including the cochlea, kidney, bladder, myometrium, adipose tissue, melanoma and esophagus. However, the yield and purity of Ti-sEVs are significantly affected by variables such as tissue dissociation strategies, enzymatic treatment conditions, and separation techniques. Despite considerable advances, the field still lacks a standardized, universally applicable protocol capable of ensuring consistent and reproducible isolation of Ti-sEVs across different tissue types and experimental settings [26].

### Advanced Ti-sEVs isolation methods

While UC, SEC, and their combination remain the dominant methods for isolating Ti-sEVs, several advanced techniques originally developed for biofluids and cell culture show significant promise for future adaptation to tissue samples.

The immunomagnetic bead method employs magnetic beads coated with specific antibodies, leveraging antigen–antibody interactions and magnetic attraction to achieve targeted adsorption and separation of EVs [34, 35]. In recent work, researchers combined the Strep-Tactin protein with the short peptide Strep-tag II to establish a Strep-tag II–based immunomagnetic separation system for EV isolation. This approach delivers higher EV yield and purity than ultracentrifugation, while markedly reducing processing time [34].

Microfluidic chip technology designs intricate microchannel networks that, together with physical, chemical, or biological mechanisms, enable efficient separation of biological particles. Moreover, these platforms can be seamlessly integrated with high-throughput analytical techniques, such as mass spectrometry and single-cell sequencing, to provide deeper insights into EV composition and function [35, 37, 38].

Asymmetric-Flow Field-Flow Fractionation (AF4) is an advanced, label-free method widely used for EV isolation and analysis. It separates particles by size and hydrodynamic properties within a laminar flow in a narrow channel coupled to a perpendicular crossflow [37]. Smaller particles are carried farther from the channel wall, while larger ones remain closer, enabling precise separation of EVs from contaminants such as protein aggregates and lipoproteins. AF4 preserves vesicle structural integrity and biological functionality, avoiding the harsh conditions often associated with UC [39, 40]. It also allows simultaneous separation of EV subpopulations for in-depth characterization. Despite these advantages, AF4 requires specialized instrumentation and expertise and often involves longer processing times; optimizing flow rates, crossflow strength, and channel dimensions is crucial to maximize yield and purity [40].

EXODUS is an innovative EV separation technology that utilizes negative pressure oscillation (NPO) and dual-coupled harmonic oscillation (HO) for efficient purification. Acting on a nano-ultrafiltration membrane, this process selectively removes free nucleic acids and proteins while retaining EVs, enabling rapid purification and enrichment. Compared with traditional methods, EXODUS offers superior speed, yield, and purity with minimal restrictions on sample volume, providing a non-invasive and cost-effective solution for simultaneous EV isolation and purification and positioning it as a promising advance for EV research and applications [41].

However, their application to the complex and viscous supernatants derived from tissue digestion remains largely unexplored, requiring method-specific optimization and rigorous validation. Further research is warranted to explore and optimize these innovative approaches for Ti-sEVs isolation, potentially addressing current limitations in yield, purity, and throughput. Following isolation, proper characterisation of Ti-sEVs is essential to confirm their identity, assess purity, and evaluate their functional relevance in cancer research.

## Characterization of Ti-sEVs

Following isolation, it is essential to properly characterize Ti-sEVs to confirm their identity, assess their purity, and evaluate their functional relevance in cancer research. While various isolation methods are being explored to address challenges in yield, purity, and throughput, the next characterization step remains crucial for understanding the biological significance of these vesicles. Ti-sEVs are defined similarly to sEVs from other sources as nano-sized, lipid bilayer–enclosed vesicular particles released by cells, typically operationally classified as populations with measured diameters below ~ 200 nm and exhibiting characteristic membrane-bound vesicular morphology by TEM or cryo-EM, while their apparent size and shape distributions remain strongly influenced by the properties of the originating tissue and the characterisation methods used.

### Morphology and size

Electron microscopy remains the gold standard for assessing vesicle structure. Scanning electron microscopy (SEM) and atomic force microscopy (AFM) typically reveal Ti-sEVs as round, cup-shaped vesicles. AFM further demonstrates that sEVs derived from malignant cells exhibit markedly reduced stiffness and adhesion, which is approximately an order of magnitude lower than their non-malignant counterparts, supporting their potential as diagnostic markers for early cancer detection and therapeutic monitoring. Transmission electron microscopy (TEM) provides even greater structural detail, showing the double-membrane architecture and a central region of low electron density characteristic of sEVs [70, 71].

Complementary sizing methods, including dynamic light scattering (DLS) and nanoparticle tracking analysis (NTA), consistently report Ti-sEV diameters in the 30–150 nm range. DLS also highlights the heterogeneity of EV populations, reflecting the complexity of the TME [72]. In metastatic melanoma, electron microscopy and NTA yielded discrepant size estimates for the sEV fraction (75 nm vs 122 nm, respectively), highlighting the need for multimodal characterization when reporting sEV size metrics [14].

### Molecular cargo

Ti-sEVs encapsulate diverse nucleic acids, including DNA, mRNA, miRNA, and other non-coding RNAs. RNA profiling distinguishes lEVs, which exhibit 18S and 28S ribosomal peaks, from sEVs that display broader small-RNA signatures and minimal ribosomal RNA [14]. In breast cancer, Ti-sEVs retained higher levels of mRNA and protein than sEVs from cell-culture models and contained 25 cancer-specific miRNAs (e.g., miR-1275, miR-1291, miR-190b-5p, miR-3178, miR-375-3p) absent from culture-derived vesicles. KEGG analysis linked these miRNAs to pathways such as endocrine resistance, adhesion, Rap1 signalling, and MAPK signalling, reinforcing their diagnostic relevance [68]. Genomic studies of malignant versus non-malignant oesophageal tissues further revealed 487 differentially expressed genes (190 up- and 297 down-regulated) enriched in PI3K–Akt, IL-17, and ECM–receptor pathways, demonstrating the capacity of Ti-sEVs to reflect tumour-specific transcriptomic landscapes [51].

Proteomic analysis of metastatic melanoma Ti-sEV subfractions showed that Ti-sEVs were enriched in EHD4, EHD1, PTGFRN, lactadherin, RAB1A, ADAM10, and ALIX, indicating that Ti-sEV cargo is molecularly heterogeneous and presence of proteins associated with membrane trafficking and EV biogenesis. Notably, ADAM10 and EHD4 were particularly enriched in the Ti-sEV fraction. In contrast, commonly used EV-associated proteins including TSG101, RAB proteins, annexins, and flotillin-1 were detected across multiple EV subpopulations, indicating limited specificity for Ti-sEV identification. In addition, PTGFRN was enriched in the same Ti-sEV fraction, accompanied by a trend toward higher abundance of CD9, CD63, and CD81, further supporting the presence of a distinct Ti-sEV-associated protein signature [13, 14].

Collectively, these morphological and molecular characteristics underscore the unique diagnostic and mechanistic insights offered by Ti-sEVs. Their ability to capture the in-situ molecular profile of the tumour microenvironment positions them as powerful tools for biomarker discovery, early cancer detection, and therapeutic monitoring.

## Biological properties of Ti-sEVs

**Fig. 2:**
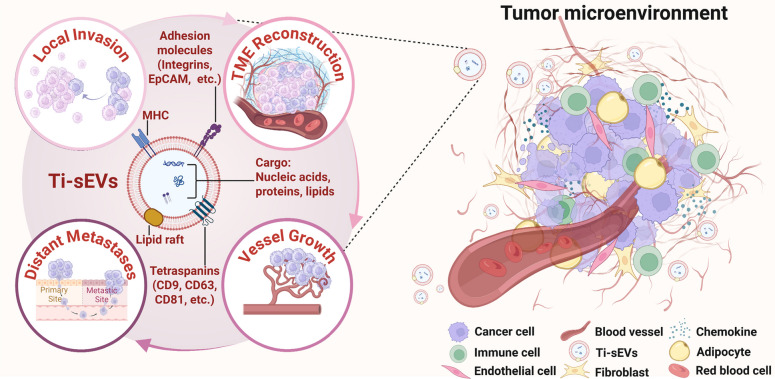
Role of Ti-sEVs in cancer progression and metastasis. Ti-sEVs, originating from the tumour microenvironment (TME), play a central role in multiple stages of cancer progression. These vesicles are enriched with cargo, including nucleic acids, proteins, and lipids, associated with their lipid raft structure. Their surface is decorated with key molecular markers, including tetraspanins (CD9, CD63, CD81, etc.), adhesion molecules (integrins, EpCAM, etc.), and major histocompatibility complex (MHC) proteins, which mediate intercellular communication and target recognition. Ti-sEVs contribute to: TME reconstruction: Ti-sEVs support the remodeling of the TME by interacting with stromal cells, such as fibroblasts and immune cells, to create a pro-tumourigenic environment. Vessel growth: They enhance angiogenesis by stimulating endothelial cells, promoting vascular growth to supply the tumour with nutrients and oxygen. Distant metastases: Ti-sEVs assist in establishing metastatic niches at distant sites by preparing recipient tissues and aiding in the dissemination of cancer cells. The figure illustrates the intricate interplay between Ti-sEVs and various cellular components within the TME, including cancer cells, immune cells, endothelial cells, fibroblasts, and glial cells, highlighting their roles in cancer progression. Local invasion: By facilitating communication between cancer cells and surrounding stromal or immune cells, Ti-sEVs promote tumour invasion into adjacent tissues. The figure illustrates the stepwise contributions of Ti-sEVs to cancer progression, highlighting their interactions with cancer cells, immune cells, endothelial cells, and fibroblasts. Abbreviations: TME, tumour microenvironment; Ti-sEVs, tissue-derived small extracellular vesicles

TME is a dynamic and intricate ecosystem comprising malignant tumour cells, tumour-infiltrating immune cells, endothelial cells, glial cells, cancer-associated fibroblasts (CAFs), and an array of cytokines and chemokines secreted by these cells [12, 73, 74]. Within the TME, sEVs from both tumour and non-tumour sources establish a sophisticated communication network that facilitates both local and systemic signaling. These sEV-mediated interactions result in a balance of pro-tumour and anti-tumour effects, influencing tumour development and progression. sEVs influence the physiological functions and metabolic states of cancer and stromal cells via autocrine, paracrine, and endocrine signaling pathways. Stromal cell-derived sEVs are particularly instrumental in shaping, sustaining, and reinforcing the TME to promote tumour progression [75, 76]. Ti-sEVs play a crucial role in various biological processes driving cancer progression, including TME remodeling, angiogenesis, local invasion, distant metastasis, and the formation of pre-metastatic niches. Figure 2 illustrates the functional roles of Ti-sEVs in the biological processes driving cancer progression within the tumor microenvironment. Within the TME, sEVs interact with fibroblasts, endothelial cells, and immune cells, influencing ECM organization and promoting angiogenesis. They also facilitate epithelial-mesenchymal transition (EMT), enhancing tumour cell migration, invasion, and metastasis. By transporting a diverse array of signaling molecules, including miRNAs, sEVs directly modulate the behavior of recipient cells, accelerating tumour growth and metastasis. These functional properties underscore the critical role of EVs in cancer progression and highlight their potential as promising therapeutic targets [77]. Moreover, the biological properties of Ti-sEVs derived from different cancer types are summarized in Table 3.

**Table 3:** Biological properties of Ti-sEVs from different tissue

Ti-sEV source	Cancer types	Cancer context	Gene(s)	Type	Brief function	References
Pancreatic tissue and lung tissue	Pancreatic and lung cancers	Tumor immune regulation/inflammation	S100A13; BSG; LGALS9	Protein	DAMP proteins enriched in tissue-derived sEVs; modulate inflammation/immune responses; promote tumor growth/metastasis; potential tumor-type markers	[29]
Colorectal tissue	Colorectal cancer (CRC)	Angiogenesis	miR-25-3p (targets KLF2/KLF4; affects VEGFR2, ZO-1, occludin, claudin-5)	RNA (miRNA)	Transferred via Ti-sEVs to endothelial cells; increases vascular permeability and angiogenesis	[78]
Colorectal tissue	CRC	Angiogenesis	GPC1; miR-96-5p; miR-149	RNA (miRNA)	GPC1 is enriched in CRC tissue sEVs and linked to vascular proliferation; miR-96-5p/miR-149 are downregulated in tumor tissue/plasma and negatively regulate GPC1	[79]
Colorectal tissue	CRC	Angiogenesis	CAT1	Protein	Overexpressed in CRC tissue sEVs; promotes endothelial proliferation and tube formation	[50]
Adipose tissue	Breast cancer	Proliferation/migration	/	/	Increase proliferation of MCF-7 and MDA-MB-231; enhance migration/invasion of MDA-MB-231	[52]
Adipose tissue	Breast cancer	Proliferation/migration/anti-apoptosis (Hippo signaling)	/	/	Internalized by MCF-7; activate Hippo signaling; promote proliferation/migration; protect against serum-deprivation/chemotherapy-induced apoptosis	[80]
Adipose tissue	Breast cancer	EMT/malignant phenotype	/	/	Induce HIF-1α and EMT, increasing growth, motility and invasiveness	[81]
Adipose tissue	Breast cancer	Metabolic reprogramming/proliferation	miR-155-5p; miR-10a-3p; miR-30a-3p	RNA (miRNA)	Enriched in adipose Ti-sEVs; enhance mitochondrial respiration and proliferation	[82]
Adipose tissue	Melanoma	Migration/invasion	/	Protein	Deliver FAO-related proteins to drive metabolic reprogramming, increasing migration/invasion	[83]
Adipose tissue	Prostate cancer	EMT/MET plasticity	/	/	Modulate EMT/MET plasticity to influence progression and metastasis	[49]

*Abbreviations: DAMP* damage-associated molecular pattern, *CRC* colorectal cancer, *EMT* epithelial-mesenchymal transition, *FAO* fatty acid oxidation, *Ti-sEVs* tissue-derived small extracellular vesicles, *MET* mesenchymal-epithelial transition

Ti-sEVs, sourced from the interstitial spaces of tumour tissues, provide a more accurate reflection of the TME than sEVs from other origins. Their distinct molecular composition has made them increasingly valuable for understanding tumour biology. Proteomic analyses of sEVs from various tumour types, such as pancreatic and lung cancers, have revealed tumour-specific proteins, including damage-associated molecular pattern (DAMP) proteins. DAMP proteins are released during cellular damage or death and play a critical role in activating the immune system and mediating inflammatory responses. Within the TME, these proteins interact with immune cells, modulating immune responses and influencing tumour progression and metastasis. A study identified DAMP proteins enriched in sEVs derived from tumour tissues, underscoring their role in tumour immune regulation. These EV-associated DAMP proteins can drive inflammatory responses and immune suppression, thereby promoting tumour growth and metastasis. Notably, specific DAMP proteins, including S100A13, BSG, and LGALS9, are highly expressed in sEVs from tumour tissues but absent in those from normal tissues. These tumour-specific DAMP proteins hold promise as biomarkers for distinguishing tumour types, providing valuable insights for cancer diagnosis and prognosis [29].

In colorectal cancer (CRC), tumour tissue cells secrete miR-25-3p, which is transferred to endothelial cells via sEVs. This transfer influences the expression of key endothelial proteins, including VEGFR2, ZO-1, occludin, and claudin-5, by targeting the transcription factors Krüppel-like factor 2 (KLF2) and Krüppel-like factor 4 (KLF4). KLF2 and KLF4 are essential regulators of angiogenesis and endothelial barrier function. Through this mechanism, miR-25-3p modulates vascular permeability and promotes angiogenesis, underscoring its pivotal role in shaping the TME [78].

Glypican-1 (GPC1), a protein highly enriched in sEVs derived from CRC tissues, belongs to the heparan sulfate proteoglycan family and is widely expressed on cell surfaces. GPC1 plays a critical role in tumour growth and angiogenesis across various malignancies [84, 85]. Notably, the expression of miR-96-5p and miR-149 is significantly reduced in the tumour tissues and plasma of patients with colorectal cancer compared with healthy controls. As these two miRNAs negatively regulate GPC1 expression, their downregulation may lead to increased GPC1 levels and suggests that CRC tissue-derived sEVs could contribute to tumour progression and angiogenic vascular proliferation, at least in part via a GPC1-dependent mechanism. [79].

Additionally, studies have revealed that high-affinity cationic amino acid transporter 1 (CAT1) is markedly enriched in sEVs derived from CRC tissues, and that EV-mediated delivery of CAT1 to the surface of vascular endothelial cells enhances arginine uptake and activates the Arg–NO–cGMP–PKG–ERK/p38 cascade, thereby stimulating endothelial proliferation and tubular structure formation and ultimately facilitating angiogenesis within the CRC microenvironment [50].

Adipose tissue, situated near various vital organs in the human body, is increasingly acknowledged as an active participant in tumour proliferation and metastasis through the secretion of EVs [86]. Emerging research indicates that adipose tissue-derived sEVs play a significant role in promoting tumourigenic processes across multiple cancer types.

In breast cancer, sEVs derived from adipose tissue were found to increase the proliferative capacity of MCF-7 and MDA-MB-231 breast cancer cell lines, while further enhancing the migratory and invasive characteristics of MDA-MB-231 cells [52]. sEVs secreted by adipocytes derived from mesenchymal stromal stem cells (MSCs) are readily internalized by MCF-7 cells, promoting their proliferation and migration via activation of the Hippo signaling pathway. These sEVs also provide protection to breast cancer cells against apoptosis induced by serum deprivation or chemotherapy drugs [80]. Additionally, sEVs from adipocytes contribute to the growth, motility, and invasiveness of breast cancer cells by inducing the HIF-1α transcription factor and driving EMT, thereby enhancing tumour malignancy [81].

Adipose Ti-sEVs were enriched with specific microRNAs, including miR-155-5p, miR-10a-3p, and miR-30a-3p, which enhance mitochondrial respiration and promote breast cancer cell proliferation [82]. Beyond breast cancer, adipose Ti-sEVs also play a critical role in other malignancies. In melanoma, they drive metabolic reprogramming by delivering proteins involved in fatty acid oxidation (FAO), significantly enhancing the migratory and invasive capacities of melanoma cells [83]. Similarly, in prostate cancer, adipose Ti-sEVs influence tumour progression and metastasis by contributing to phenotypic plasticity through the regulation of EMT and mesenchymal-epithelial transition (MET) signaling pathways [49].

## Applications of Ti-sEVs in cancer diagnosis and prognosis

Recent advancements in genomic, transcriptomic and proteomic technologies have uncovered the potential of Ti-sEVs as highly specific biomarkers and therapeutic targets in cancer research. Ti-sEVs, unlike their plasma- or serum-derived counterparts, often exhibit greater molecular specificity, offering enhanced diagnostic accuracy and novel insights into tumour biology. This review explores the multifaceted roles of sEVs across diverse cancer types, emphasizing their diagnostic, prognostic, and therapeutic potential. By highlighting key studies across various cancers, including esophageal, renal, colorectal, ovarian, and glioblastoma, this review aims to provide a comprehensive understanding of the role of Ti-sEVs playing in cancer diagnosis and prognosis. The roles of Ti-sEVs in the diagnosis and prognosis of various cancers are summarized in Table 4.

**Table 4:** The roles of Ti-sEVs in cancer diagnosis and prognosis

Ti-sEVs source	Ti-sEVs isolation	Ti-sEVs characterization	sEVs size	Ti-sEVs Analysis method	Highlights	References
Esophageal tissue	Size-Exclusion Chromatography(SEC) Ultrafiltration(UF)	Western blot NanoFCM TEM	50–200 nm (nFCM); < 120 nm (TEM)	proteomic sequencing	The CD54 protein is highly expressed in esophageal cancer tissues and has diagnostic potential; silencing CD54 can inhibit cancer cell proliferation and migration	[87]
Clear Cell Renal Cell Carcinoma (ccRCC) tissue	Ultracentrifugation (UC) Density gradient centrifugation	WB NTA TEM	36–120 nm (TEM)	Immunohistochemistry	The expression levels of CA9, CD70, and CD147 proteins in tumour tissue EVs are significantly elevated, showing potential as diagnostic biomarkers for liquid biopsies in ccRCC	[62]
Colorectal tissue	UC	WB TEM	< 200 nm (TEM)	Liquid Chromatography-Mass Spectrometry ELISA Metabolomic analysis	The CAT1 protein found in tissue EVs is highly expressed in the plasma of CRC patients, showing potential for diagnosing CRC, while EVs with overexpressed CAT1 mediate tumour angiogenesis	[50]
Bladder cancer tissue	SEC	Nano-flow Cytometry (nFCM) TEM WB	50–150 nm (nFCM)	transcriptome RNA sequencing	Four sEV-related bladder cancer-specific mRNA biomarkers (FAM71E2, OR4K5, FAM138F, and KRTAP26-1) were identified, holding promise for use in liquid biopsies	[63]
Ovarian cell carcinoma (OCC) tissue	UC	TEM WB	NR	miRNA Microarray Analysis Quantitative Reverse Transcription-Polymerase Chain Reaction (qRT-PCR)	Six miRNAs, including miR-200a-3p, miR-200b-3p, miR-200c-3p, miR-200a-5p, and miR-200b-5p, significantly decreased in the serum samples of postoperative OCC patients and can be used for the early diagnosis and disease monitoring of OCC	[66]
ccRCC tissue	UC Density gradient centrifugation	TEM WB NTA	30–200 nm (TEM/NTA)	Flow Cytometry (FACS) RNA Sequencing Immunoblot Analysis	PD-L2 in sEVs as a mediator of immune escape in ccRCC and suggests that targeting PD-L2 on sEV could be a promising therapeutic approach	[48]
Adipose tissue	ExoQuick-TCTM precipitation reagent	NTA Protein Expression	100–200 nm (NTA)	ELISA quantitative real-time PCR	adipose tissue-derived sEVs from obese mice may play a role in suppressing splenocyte-mediated Panc02 cell death and potentially drive adverse effects of cancer immunotherapy through the upregulation of IL-4, IL-2, and IL-12p70	[88]
Adipose tissue	SEC	Multiplex EV Marker Assay by using a 6-Plex Human ProcartaPlex Panel on a Luminex 200 device NTA DLS	50–150 nm (NTA)	real-time PCR In Vivo Model (Chicken Embryo Chorioallantoic Membrane—CAM)	ASC-EVs possess antitumour properties, reducing GBM cell proliferation and invasiveness, and can be applied as anticancer therapeutics and medicine carriers	[89]
Oral squamous cell carcinoma tissue	UC	TEM WB NTA	131 nm (NTA)	Flow Cytometry ELISA Mass Spectrometry Immunohistochemistry	The spatial distribution of Ti-sEVs can serve as a predictive indicator for tumour recurrence, and the characteristics of Ti-sEVs can predict patients' prognosis in response to anti-PD-1 immunotherapy	[64]
Colorectal tissue	UC Density gradient centrifugation	TEM WB NTA	30–200 nm (TEM)	proteomic sequencing	The significant expression levels of four proteins (HLA-DPA1, S100P, NUP205, PCNA) in the adjacent tissues of the relapsed group can be used to predict the risk of relapse in postoperative follow-ups	[67]
Epithelial ovarian cancer (EOC) tissue	UC SEC	TEM WB NTA	30–200 nm (NTA)	proteomic sequencing	Proteomics and LASSO regression analysis identified key immune-related proteins (IRPs) and constructed a predictive model capable of forecasting the efficacy of platinum-based chemotherapy in patients with EOC	[65]
Adipose tissue	UC	TEM WB NTA Nano-flow Cytometry	65.6–69.6 nm (nFCM)	RT-PCR	High MTTP expression in plasma sEVs of CRC patients with a high body fat ratio was associated with an inhibitory effect on ferroptosis, reducing sensitivity to chemotherapy	[90]

*Abbreviations: DNase* deoxyribonuclease, *ELISA* enzyme-linked immunosorbent assay, *FACS* flow cytometry, *IRPs* immune-related proteins, *LASSO* least absolute shrinkage and selection operator, *miRNA* microrna, *MTTP* microsomal triglyceride transfer protein, *nFCM* nano-flow cytometry, *NR* not reported, *NTA* nanoparticle tracking analysis, *PBS* phosphate-buffered saline, *qRT-PCR* quantitative reverse transcription-polymerase chain reaction, *SEC* size exclusion chromatography, *TEM* transmission electron microscopy, *UC* ultracentrifugation, *UF* ultrafiltration, *WB* western blot

Hoshino et al. identified a set of Ti-sEVs proteins that hold potential as diagnostic biomarkers for cancer through an analysis of sEVs proteins in tumour tissues and plasma. The study revealed a low correlation between the protein profiles of plasma- or serum-derived sEVs and those derived directly from tissues. This finding suggests that plasma-derived sEV molecules may lack specificity, emphasizing the advantages of identifying biomarkers from Ti-sEVs for improved diagnostic accuracy [29].

In a study on esophageal cancer, 803 differentially expressed proteins were identified in sEVs extracted from esophageal cancer tissues and adjacent normal tissues, with 686 proteins upregulated and 117 downregulated. Among these, the expression level of sEVs CD54 protein in plasma samples from 122 esophageal cancer patients was significantly higher than that in healthy controls and correlated with disease progression. Receiver operating characteristic analysis showed that plasma exosomal CD54 achieved an area under the curve (AUC) of 0.702, with 66.13% sensitivity and 71.31% specificity for distinguishing esophageal cancer patients from healthy individuals, whereas its performance for stage I disease was modest (AUC 0.513, sensitivity 32.26%, specificity 52.38%). Functional studies demonstrated that silencing CD54 significantly inhibited the proliferation and migration of esophageal cancer cells, suggesting that CD54 is not only a potential diagnostic biomarker for esophageal cancer but also plays a role in disease progression. This study highlights Ti-sEVs potential utility in early diagnosis and therapeutic strategies, offering a promising new direction for managing esophageal cancer [87].

In bladder cancer, proteomic profiling integrated urinary EVs with Ti-sEV related sEVs, specifically tissue-exudative EVs (Te-EVs), which are released into serum-free medium during short-term ex vivo culture of freshly resected tumour tissue, to nominate tumour-enriched membrane proteins. In a validation cohort of 40 patients and 30 healthy controls, urinary EV-associated HSP90, SDC1 and MARCKS were significantly increased, with HSP90 showing the best diagnostic performance (AUC 0.813, 82.5% sensitivity, 70.0% specificity), all exceeding urine cytology. These data support Te-EV–guided screening for clinically relevant urinary EV biomarkers [91].

Clear Cell Renal Cell Carcinoma (ccRCC) is the most common subtype of Renal Cell Carcinoma (RCC), comprising 70% to 80% of all kidney cancer cases. Researchers isolated sEVs from ccRCC cell lines (786-O, RCC53, Caki1, and Caki2) and patient tissue samples, detecting significantly higher expression levels of proteins CA9, CD70, and CD147 in tumour tissue lysates and sEVs compared to normal tissues. These proteins show promise as potential diagnostic biomarkers for liquid biopsy in ccRCC, offering new avenues for non-invasive cancer detection and monitoring [62].

Colorectal Cancer, the third most prevalent cancer worldwide, has been studied using SEC to isolate sEVs from colorectal tumour tissues and adjacent normal tissues. Analysis revealed that the CAT1 protein in Ti-sEVs was significantly overexpressed in CRC tissues, indicating its potential as a biomarker for early disease diagnosis [50].

Research on Ti-sEVs in cancer diagnosis extends beyond proteomics. In bladder cancer studies, researchers isolated sEVs from various sources, including whole blood, urine, fresh tissue samples, and formalin-fixed paraffin-embedded (FFPE) tumour tissues, and performed comprehensive transcriptomic RNA sequencing. The analysis revealed a high consistency in molecular subtype classification between FFPE tumour tissues and sEVs derived from tumour tissues.

Comparative analysis of sEVs from urine and FFPE tumour tissues in non-muscle-invasive bladder cancer (NMIBC) and muscle-invasive bladder cancer (MIBC) revealed shared activation of key signaling pathways. Furthermore, a comparative analysis of sEVs from urine and tumour tissues with those from adjacent normal tissues identified four potential bladder cancer-specific sEVs mRNA biomarkers. These findings underscore the potential of sEVs transcriptomic profiling in advancing bladder cancer diagnostics and subtype classification [63].

In a study on ovarian cancer, researchers conducted miRNA microarray analysis on sEVs isolated from ovarian clear cell carcinoma (OCC) tissues and normal ovarian tissues, identifying 37 miRNAs that were upregulated in OCC. Subsequent qRT-PCR validation revealed that six miRNAs including miR-200a-3p, miR-200b-3p, miR-200c-3p, miR-200a-5p, miR-200b-5p, and miR-30a-5p, showed significant decreases in serum samples from OCC patients after surgery. Notably, these miRNAs did not exhibit significant changes in patients with atypical endometrial hyperplasia (AEH). These findings indicate that the identified miRNAs hold promise as biomarkers for early diagnosis and disease monitoring in OCC, emphasizing their potential clinical applicability [66].

The progression and invasion of malignant tumours are major factors contributing to poor prognosis, with Ti-sEVs playing a critical role in cancer advancement. Programmed death-ligand 2 (PD-L2), which interacts with programmed death receptor 1 (PD-1), has been identified as a significant mediator in this process. In studies on renal clear cell carcinoma, researchers discovered that PD-L2 protein is predominantly located in the intercellular spaces, with the highest concentration observed on the surface of EVs in these regions. Interestingly, the expression level of tumour-derived extracellular PD-L2 (TDE-PD-L2) surpasses that of tumour-derived extracellular PD-L1 (TDE-PD-L1) in various cancers. Functionally, TDE-PD-L2 exhibits dual effects depending on the immune context. In the absence of adaptive immunity, tumour-derived extracellular PD-L2 (TDE-PD-L2) suppresses tumour growth and metastasis. However, in the presence of an intact immune system, TDE-PD-L2 binds to immune cells via PD-1, increasing the proportion of regulatory T cells while reducing cytotoxic CD8 + T cells. This interaction systematically impairs the function of tumour-infiltrating T cells and peripheral T cells in the spleen, contributing to immune suppression. This immune modulation promotes tumour growth and metastasis, ultimately impacting disease progression [48].

Studies have shown that Ti-sEVs derived from the adipose tissue of obese mice significantly decreased the mortality of Panc02 pancreatic cancer cells induced by splenocytes. This finding suggests that these sEVs may inhibit the immune system's ability to target and eliminate pancreatic cancer cells. Additionally, the sEVs significantly reduced the expression of the SMAD4 gene in Panc02 cells. As a known tumour suppressor, reduced SMAD4 expression is likely linked to cancer progression [92], highlighting the potential role of adipose Ti-sEVs in modulating tumour-immune interactions and promoting tumourigenesis [88].

Glioblastoma multiforme (GBM), an aggressive central nervous system tumour, is associated with an extremely poor prognosis. Recent studies revealed that sEVs derived from adipose tissue mesenchymal stem cells (ASC-EVs) markedly inhibited GBM cell proliferation. Additionally, ASC-EVs downregulated the expression of invasion-related genes ITGα5 and ITGβ3, along with the angiogenesis-promoting gene KDR, thereby effectively suppressing GBM progression [89].

The therapeutic potential of Ti-sEVs is increasingly being explored. In studies on oral squamous cell carcinoma (OSCC), researchers performed multi-regional sampling of OSCC tissues to investigate the spatial distribution of Ti-sEVs, tumour margins, and peritumoural tissues. The findings revealed that the accumulation of Ti-sEVs in peritumoural areas, including tumour margins and surrounding tissues, was associated with a higher disease-free survival rate. Specifically, a higher quantity of Ti-sEVs in these areas correlated with a reduced risk of recurrence, whereas lower Ti-sEV levels indicated a higher risk of tumour recurrence. This suggests that the spatial distribution of Ti-sEVs could serve as a predictive indicator for tumour recurrence. In advanced OSCC patients, the quantity and spatial distribution of Ti-sEVs prior to anti-PD-1 therapy showed a strong correlation with clinical outcomes, indicating their potential as predictive biomarkers for immunotherapy response. Moreover, temporal variations in Ti-sEVs before and after treatment were associated with clinical responses, highlighting the value of monitoring these changes to assess treatment effectiveness. The study further highlights that the characteristics of Ti-sEVs can help distinguish patients experiencing tumour shrinkage from those with tumour progression. These findings underscore the potential of Ti-sEVs as biomarkers for predicting treatment response and offer a promising avenue for personalized therapy in cancer management [64].

In CRC research, sEVs derived from CRC tissues have been found to carry significant levels of four proteins: HLA-DPA1, S100P, NUP205, and PCNA. These proteins have shown potential as biomarkers for predicting the risk of recurrence during postoperative follow-up and for assessing treatment efficacy [67].

Epithelial Ovarian Cancer (EOC) is a highly lethal malignancy often characterized by resistance to platinum-based chemotherapy. In a recent study, researchers isolated Ti-sEVs from 58 platinum-sensitive and 30 platinum-resistant EOC patients and conducted 4D data-independent acquisition (DIA) proteomic sequencing. Using LASSO regression analysis, they identified key immune-related proteins (IRPs) and constructed a predictive model capable of forecasting the efficacy of platinum-based chemotherapy in EOC patients. This model offers a promising tool for personalized treatment strategies in managing EOC [65]. Ti-sEVs play a critical role in cancer drug resistance. In CRC, sEVs originating from adipose tissue have been shown to reduce sensitivity to ferroptosis, thereby enhancing chemoresistance to oxaliplatin. These findings highlight the influence of tissue-derived sEVs in mediating resistance to cancer therapies [90].

The burgeoning field of EV research has illuminated the pivotal role of Ti-sEVs in cancer biology, providing unprecedented opportunities for non-invasive diagnostics, therapeutic interventions, and personalized medicine. Studies have demonstrated the unique molecular signatures of Ti-sEVs across various cancer types, revealing their potential as precise biomarkers for early detection, prognosis, and treatment monitoring. Moreover, the functional contributions of Ti-sEVs to tumour progression, immune evasion, and chemoresistance underscore their dual utility as both diagnostic tools and therapeutic targets. Innovations in multi-omics approaches and bioengineering of sEVs, such as combining synthetic vesicles with tumour-derived sEVs, have further expanded their therapeutic potential, offering safer and more effective strategies for cancer immunotherapy. Despite these advancements, challenges remain in standardizing EV isolation, characterization, and clinical application. Future research should focus on addressing these limitations to fully harness the promise of Ti-sEVs in transforming cancer diagnostics and treatment paradigms. Together, these findings highlight the central role of Ti-sEVs in bridging the gap between fundamental research and clinical oncology, paving the way for more effective and tailored cancer management strategies.

## Current challenges for Ti-sEVs in cancer research

Ti-sEVs hold significant promise in cancer research but face substantial challenges that currently limit their full application. The isolation of Ti-sEVs is particularly complex due to their heterogeneity and the difficulty in separating them from cellular debris, protein aggregates, and other contaminants. Each pretreatment method involves a trade-off among yield, purity, and the risk of tissue damage or contamination, and the absence of standardized, universally accepted protocols leads to inconsistencies across studies. This lack of reproducibility undermines the development of robust biomarkers and reliable clinical applications. In terms of separation methods, conventional techniques, such as UC and SEC, often yield mixed populations, thereby compromising downstream molecular and functional analyses. Additionally, the lack of robust, high-throughput characterization methods further hinders progress in this field. Recent methodological work suggests that optimizing tissue processing according to tissue type may help improve Ti-sEV isolation. For example, a gentler protocol using mild enzymatic dissociation, tissue-specific buffers, DNase-assisted reduction of debris clumping, and density-gradient purification was reported to reduce intracellular and non-vesicular contamination while preserving Ti-sEV integrity [93].

A significant challenge in the field is the lack of standardized, universally accepted protocols for Ti-EVs isolation and characterization. Differences in sample processing, storage conditions, and analytical methodologies result in inconsistencies across studies, compromising reproducibility and hindering the development of reliable biomarkers and clinical applications. Accordingly, improved standardization should be pursued through transparent reporting of tissue source, storage status, dissociation conditions, pre-clearing steps, and purification workflows, which may improve cross-study comparability and reproducibility [94].

The purification of EVs from tissue samples is especially challenging due to the complexity of tissue matrices. Non-vesicular components, such as lipoproteins, soluble proteins, and organelles, frequently co-isolate with Ti-sEVs, resulting in contamination that compromises the accuracy of molecular and functional studies. Looking forward, more advanced downstream separation and purification strategies may help refine post-dissociation Ti-sEV preparations. In particular, immunoaffinity capture, AF4-based fractionation, and rapid membrane- or microfluidics-based platforms exemplified by EXODUS have been highlighted as promising approaches to improve purity, analytical specificity, and in some settings scalability, although direct validation in solid-tissue EV workflows remains limited and most supporting evidence still derives from liquid-sample studies [94–97].

Although advances in multi-omics approaches, including transcriptomics, proteomics, and lipidomics, have enhanced our understanding of EV biology, significant gaps remain in elucidating the molecular and functional roles of Ti-sEVs in cancer. Their involvement to tumour progression, immune modulation, and metastasis are not yet fully understood, limiting their utility as diagnostic tools or therapeutic targets. Recent proteomic studies have also identified candidate tissue-EV markers that may improve source tracing, while emerging EV–organoid/3D culture frameworks may provide more physiologically relevant systems for mechanistic studies when direct tissue workflows are technically constrained [98, 99].

Overall, addressing these challenges will require improved standardization, contamination-aware isolation workflows, and closer integration of molecular profiling with functional validation.

## Conclusions and future perspectives

Ti-sEVs have emerged as a growing area of interest in EV research in cancer, offering distinct advantages over EVs derived from body fluids and culture media. These include reduced exogenous contamination and a more accurate representation of the in vivo TME. However, several obstacles must be overcome to unlock their full potential.

First, the lack of standardized and universally accepted isolation methods for Ti-sEVs remains a critical barrier. Intracellular vesicle contamination often compromises sample purity, necessitating the development of specific and standardized isolation techniques tailored to the heterogeneity of human tissues. Second, achieving diagnostic precision at the single-EV level requires extensive large-scale clinical trials across diverse cancer tissues to identify universal or tumour-specific EV markers. Third, the inherently low yield of Ti-sEVs from individual samples poses challenges for reproducibility and consistency in follow-up studies. Additionally, concerns remain about whether extended culture durations alter the properties of EVs derived from isolated tissue cultures. Finally, the role of Ti-sEVs in cancer diagnosis and their involvement in pathophysiological processes requires further investigation to clarify their mechanisms and applications.

This review discusses the concept, advantages, extraction methods, and applications of Ti-sEVs in the context of cancer diagnosis and prognosis. While current isolation methods are advancing, significant limitations persist. Ti-sEVs are becoming increasingly recognized as a valuable tool in oncology research due to their higher purity and precise reflection of tissue-specific environments. Current approaches often integrate Ti-sEVs with sEVs derived from cancer cell lines or human body fluids to screen for cancer-specific markers. With continued advances in standardizing isolation and purification techniques, Ti-sEVs hold considerable promise for cancer diagnosis and prognosis. Looking forward, the clinical translation of Ti-sEVs will likely depend less on further increasing methodological complexity alone and more on the establishment of tissue-specific, standardized, and transparently reported workflows that are reproducible across laboratories and compatible with clinical sampling constraints. A particularly promising translational strategy is to use Ti-sEVs as a tissue-level discovery platform for identifying tissue-associated EV markers, followed by validation of these candidate signals in accessible body fluids. This strategy effectively links the biological specificity of tissue-derived vesicles with the practicality of liquid biopsy [100]. In parallel, future progress will also require more reliable source-tracing strategies and improved model systems, such as organoid-based EV platforms, to better bridge the gap between in situ tissue biology and clinically scalable biomarker development [101]. Collectively, these advances may enable Ti-sEVs to evolve from a mainly exploratory research tool into a more clinically actionable framework for cancer diagnosis, prognosis, and precision monitoring.

## Data Availability

No datasets were generated or analysed during the current study.

## Abbreviations

AEH: Atypical endometrial hyperplasia
AF4: Asymmetric-flow field-flow fractionation
AFM: Atomic force microscopy
ASC-EVs: Adipose tissue mesenchymal stem cells
BF-sEVs: Body fluid-derived sEVs
CAFs: Cancer-associated fibroblasts
CAT1: Cationic amino acid transporter 1
CCM-sEVs: Cell culture medium-derived sEVs
CRC: Colorectal cancer
ccRCC: Clear cell renal cell carcinoma
DAMP: Damage-associated molecular pattern
DIA: Data-independent acquisition
DLS: Dynamic light scattering
EOC: Epithelial ovarian cancer
ECM: Extracellular matrix
EMT: Epithelial-mesenchymal transition
EVs: Extracellular vesicles
FFPE: Formalin-fixed paraffin-embedded
FAO: Fatty acid oxidation
GPC1: Glypican-1
HO: Harmonic oscillation
IRPs: Immune-related proteins
KLF2: Krüppel-like factor 2
KLF4: Krüppel-like factor 4
lEVs: Large extracellular vesicles
MET: Mesenchymal-epithelial transition
MIBC: Muscle-invasive bladder cancer
MSCs: Mesenchymal stromal stem cells
NMIBC: Non-muscle-invasive bladder cancer
NPO: Negative pressure oscillation
NTA: Nanoparticle tracking analysis
OSCC: Oral squamous cell carcinoma
OCC: Ovarian clear cell carcinoma
PD-1: Programmed death receptor 1
PD-L2: Programmed death-ligand 2
RCC: Renal cell carcinoma
SEC: Size exclusion chromatography
SEM: Scanning electron microscopy
TEM: Transmission electron microscopy
Te-EVs: Tissue-exudative extracellular vesicles
Ti-EVs: Tumour-derived extracellular vesicles
Ti-sEVs: Tissue-derived small extracellular vesicles
TME: Tumour microenvironment
UF: Ultrafiltration
UC: Ultracentrifugation
WB: Western blot
